# Multiplex qPCR facilitates identification of betaherpesviruses in patients with acute liver failure of unknown etiology

**DOI:** 10.1186/s12879-019-4309-4

**Published:** 2019-09-04

**Authors:** Jéssica Vasques Raposo, Arthur Daniel Rocha Alves, Alexandre dos Santos da Silva, Damião Carlos dos Santos, Juliana Gil Melgaço, Otacílio C. Moreira, Marcelo Alves Pinto, Vanessa Salete de Paula

**Affiliations:** 1 Laboratory of Molecular Virology, Oswaldo Cruz Institute / Fiocruz, Rio de Janeiro, Brazil; 2 Laboratory of Technological Development in Virology, Oswaldo Cruz Institute / Fiocruz, Rio de Janeiro, Brazil; 3 Laboratory of Molecular Biology and Endemic Diseases, Oswaldo Cruz Institute / Fiocruz, Rio de Janeiro, Brazil; 4 Estácio de Sá University, Rio de Janeiro, Brazil; 5 Oswaldo Cruz Foundation, IOC – Av. Brasil 4365-Manguinhos, Pav. Helio e Peggy Pereira B10, Rio de Janeiro, 21040-360 Brazil

**Keywords:** Betaherpesviruses, Acute liver failure, qPCR, Multiplex, Liver

## Abstract

**Background:**

The etiology of acute liver failure (ALF) is often unknown and reported to be associated with herpesviruses in a number of cases. In this study, we examined for betaherpesviruses infections in patients with ALF of unknown etiology using a multiplex qPCR to Betaherpesviruses subfamily.

**Methods:**

Liver explant and serum samples from 27 patients with ALF of unknown etiology were analyzed with the aid of multiplex qPCR to identify betaherpesviruses. All positive samples were sequenced to confirm herpes infection and liver enzyme levels evaluated.

**Results:**

Betaherpesviruses infection was effectively detected using multiplex qPCR. Six (22%) HHV-6, one (3%) HCMV and two (7%) dual infections (one with HHV-7/HHV-6, and the other with HHV-7/ HCMV). Interestingly, HHV-7 was only detected in the presence of other betaherpesviruses. Sequencing information confirmed betaherpesviruses infection. High hepatic enzyme levels and INR values> 1.5 were determined in all betaherpesvirus-positive patients.

**Conclusions:**

Multiplex qPCR facilitated efficient quantification, indicating that differentiation between betaherpesviruses is possible with the sole use of real-time PCR. Liver explant and serum samples were positive for some betaherpesviruses, and coinfection of HHV-7 with HHV-6 and HCMV was additionally detected. Based on these results, we propose that ALF patients should be screened for the presence of betaherpesviruses.

## Background

Acute liver failure (ALF) is a condition whereby healthy liver deteriorates rapidly, resulting in jaundice, encephalopathy and coagulopathy. According to the recently stablished diagnostic criteria, ALF may be defined when the interval between the appearance of jaundice and the development of coagulopathy and encephalopathy is up to 8 weeks [1]. The etiology of ALF cases is often unknown and some cases are not positive for hepatotropic viruses or other established potential factors [2]. Previous studies have reported the presence of viruses of the family *Herpesviridae* in cases of ALF, including the betaherpesviruses human cytomegalovirus (HCMV), human herpesvirus 6 (HHV-6), and human herpesvirus 7 (HHV-7) [3, 4].

HCMV triggers an infection similar to mononucleosis syndrome while HHV-6A/B and HHV-7 cause *roseola infantum*, febrile seizures and other febrile syndromes in children [4, 5]. After infection, betaherpesviruses remain latent until conditions in the immunocompromised host favor reactivation. HHV-6A/B and HHV-7 display genetic and biological homology and commonly manifest as sudden rash [6]. Primary HCMV and/or HHV-6A/B infections can cause mild and self-limiting hepatitis in immunocompetent patients. However, in immunocompromised cases, in particular, organ transplant recipients, HCMV infection is associated with increased morbidity [7]. Primary infection with HHV-6 is a frequent cause of ALF, necessitating antiviral therapy during early post-transplantation procedures. A number of studies have disclosed the presence of HHV-6 in liver samples from pretransplant cases [8, 9]. Complications resulting from HHV-7 infection have also been identified as a risk in organ transplantation [10]. On the other hand, although have been rarely described, there are increasing evidences of association between some betaherpesviruses and ALF in immunocompetent patients [11–13].

Poor prognosis of hepatitis evolving from herpesvirus infection is associated with late diagnosis and delayed specific antiviral therapy, highlighting the urgent need for early detection. Currently, while several protocols exist for identification of the herpesvirus genome [14],multiple PCR assays are required for the process. In recent years, real-time PCR has been increasingly applied to simplify the overall procedure and reduce performance time in detecting and quantifying virus in clinical samples [15, 16]. Multiplex qPCR has been suggested as an efficient alternative for simultaneous detection of HCMV, HHV-6 and HHV-7 within the same reaction [9, 17]. In the current study, the incidence of betaherpesvirus infection in patients with ALF of unknown etiology was investigated using the multiplex qPCR approach.

## Methods

### Patients and samples

The study group consisted of 27 patients with clinical signs of ALF (24 females and 3 males), who were attended at the hepatic transplantation service from Federal Hospital of Rio de Janeiro, Brazil. The protocol was approved by the Ethics Committee of the Oswaldo Cruz Institute (Protocol no. 440.614) and the written informed consent was signed by the patients or the responsible person. Inclusion criteria demanded the development of coagulopathy [i.e. prothrombin time activity (PTA) > 15 s or international normalized ratio (INR) > 1.5] and hepatic encephalopathy within 8 weeks of jaundice onset in the absence of pre-existing liver disease. The clinical and haematological data of these patients, such as PTA, INR values and total bilirubin (TB) were obtained from medical records during the intensive care period. Exclusion criteria were any histological feature of chronic disease such as fibrosis.

Peripheral blood and liver explant samples were collected during the liver transplantation procedures. Peripheral blood were collected just before the hepatectomy procedure in trisodic citrate dextrose solution A/ACD-A (Greiner Bio-one, Kremsmünster, Austria). Fragments of liver explant were collected just after hepatectomy inside surgery center and immediately snap frozen in liquid nitrogen. As some patients died before liver transplantation, was not possible obtain liver explant samples of all patients. Thus, peripheral blood for testing were obtained from all patients and liver explant acquired from 14 patients. Samples had been previously determined as negative for hepatitis A, B, C, E and of unknown etiology.

Samples were examined via real-time multiplex PCR and confirmed using singleplex, qualitative PCR and nucleotide sequencing.

### Extraction of DNA

Viral DNA was extracted using the QIAamp DNA Blood Mini Kit for serum samples and QIAamp DNA Tissue Mini Kit for liver samples (Qiagen, Germany), according to the manufacturer’s protocols. DNA samples were stored at − 70 °C until processing.

### Multiplex qPCR for betaherpesviruses

Betaherpesviruses were detected and quantified according to the method of Sassenscheidt and colleagues [18]. Oligonucleotide probes for detection of betaherpesviruses target highly conserved regions in the viral genome of published strains. For amplification of HCMV, we used UL54 and the U56 regions were used for HHV-6 A/B. This assay does not allow differentiation between consensus HHV-6 types A and B in clinical samples [18]. For detection of HHV-7, the U37 region were used as described earlier [19]. Primers and oligonucleotide TaqMan probes are presented in Table 1. Multiplex qPCR was performed in a reaction mixture comprising 1 μL 25x PCR Enzyme (Mix Life Technologies, California, USA), 2.5 μL of each oligonucleotide (3 μM), 2 μL probe (0.4 μM), 12.5 μL of 1x PCR Buffer (Life Technologies, California, USA) and 5 μL DNA. Absolute quantification of DNA virus was performed with the aid of a synthetic standard curve designed for this study, ranging from 5 to 5 × 10^8^ genome copies/uL (Table 1).

**Table 1:** Sequences of primers, probes and standard curves

	HCMV Sequence (5′-3′)	HHV-6Sequence (5′-3′)	HHV-7Sequence (5′-3′)
	FAM-	VIC-	NED –
Probes	CCGTATTGGTGCGCGATCTGTTCAA- NFQ-MGB	TTAGATGGTGGTGAGCTGGGATCGGT- NFQ-MGB	CTCGCAGATTGCTTGTTGGCCATG- NFQ-MGB
Primers (Sense)	GGCCGTTACTGTCTGCAGGA	AAAGACCTAAATTGCCGCTACCT	CGGAAGTCACTGGAGTAATGACAA
Primers (Anti-sense)	GGCCTCGTAGTGAAAATTAATGGT	GCAAGCTCATGAACATCGTCA	CCAATCCTTCCGAAACCGAT
Standard Curve	TTCGTGGCCTCGTAGTGAAAATTAATGGTCGTATTTGAACAGATCGCGCACCAATACGGATGCGTTCCTGCAGACAGTAACGGCCCTGATA	TTCGTGCAAGCTCATGAACATCGTCACGTATACCGATCCCAGCTCACCACCATCTAAATGCGTAGGTAGCGGCAATTTAGGTCTTTCTGATA	TTCGTCCAATCCTTCCGAAACCGATCGTATCATGGCCAACAAGCAATCTGCGAGATGCGTTTGTCATTACTCCAGTGACTTCCGCTGATA

### Singleplex qPCR for betaherpesviruses

We employed the protocol described by Sassenscheidt et al. [18] using the same primers, oligonucleotide probes and amplification regions. For each reaction, a mixture comprising 1 μL 25x PCR Enzyme (Mix Life Technologies, California, USA), 2.5 μL each oligonucleotide (1 μM), 2.0 μL probe (0.4 μM) and 12.5 μL of 1x PCR Buffer (Life Technologies, California, USA) was used according to the manufacturer’s instructions. Absolute quantification was performed based on the synthetic standard curve designed for this study.

### Specificity analysis

Different targets of the Herpesviridae family were used for analysis of specificity. Serum samples positive for herpes simplex virus 1 and 2 (HSV-1 and HSV-2) and Epstein-Barr virus (EBV) were examined via previously standardized multiplex standard qPCR using betaherpesvirus targets as the negative control.

### Reference control

The TaqMan RNase P Control Reagent kit (Applied Biosystems Foster City, USA, Catalog number 4316844) was used as an internal reference control for analysis of human clinical samples to ensure the quality of DNA and exclude the possibility of false-negative results due to the presence of PCR inhibitors or low DNA integrity. TaqMan RNase P probe was 5′-labeled with VIC fluorophore and 3′-labeled with NFQ-MGB. The reference control was used in qPCR singleplex assays and quality of samples monitored based on Ct values. The reaction mixture for RNase P control comprised 6 μL DNase/RNase-free water, 1 μL of 25X qPCR enzyme mix, 12.5 μL of 2X qPCR buffer (including the reference dye ROX) and 1 μL RNase P mix (containing oligonucleotides for this region and specific buffers; Applied Biosystems, California, USA) in 96-well plates. In addition, 5 μL extracted DNA was subsequently added to the mix on the plate under similar conditions to those used in qPCR for betaherpesvirus detection.

### Qualitative Pan herpesvirus-PCR

Samples showing positivity in qPCR were additionally examined via qualitative Pan-PCR only to confirm diagnosis. For simultaneous detection of betaherpesviruses, we employed the Pan herpesvirus technique based on amplification via nested PCR (Pan-PCR) of the *Dpol* gene, a highly conserved region in the herpesvirus genome, and species identification via sequencing of the viral genome. The protocol for herpesvirus detection and specific oligonucleotides were designed based on the report by Ehlers et al. [2].

### Sequencing

Reactions were conducted in the DNA sequencing platform of the Technological Development Program in Health Supplies /PDTIS (Oswaldo Cruz Foundation, Rio de Janeiro, Brazil). Nucleotide sequences obtained were analyzed in the BioEdit 7.2.5 program and compared to other sequences deposited in GenBank with the BLAST tool (Basic Local Alignment Search Tool) to identify the betaherpesvirus species detected using nested PCR.

### Biochemical analysis of samples

Biochemical analyses were performed via spectrophotometric determination of pyruvic transaminase (ALT), oxaloacetic transaminase (AST) and alkaline phosphatase. Spectrophotometry procedures were performed using commercial kits (Abbott, Illinois, United States of America). For analysis of biochemical markers, samples were divided into three groups: betaherpesvirus-positive ALF samples (ß-Herpesvirus positive), betaherpesvirus-negative ALF samples (ß-Herpesvirus negative) and healthy controls. Diagnosis of ALF was performed based on grade of encephalopathy along with prothrombin time (PT) and international normalized ratio (INR).

## Results

### Detection and quantification of betaherpesviruses

**Fig. 1:**
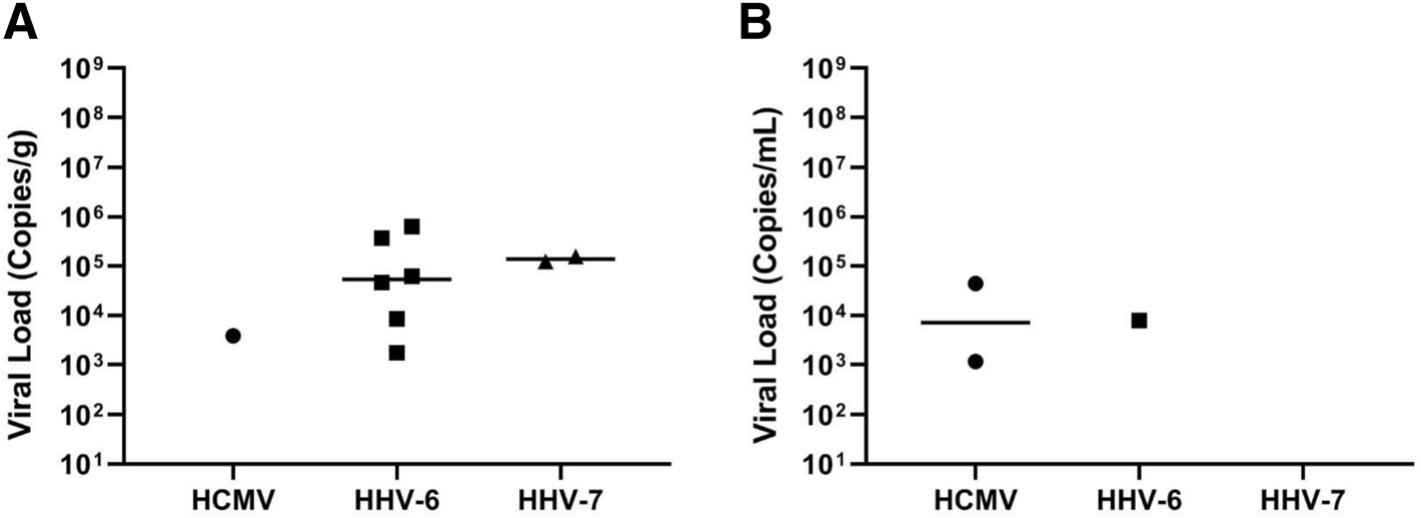
Comparison of viral loads (copies/g) in liver (A) and serum (B) from patients with ALF of unknown etiology positive for betaherpesviruses, specifically, human cytomegalovirus (HCMV), human herpesvirus 6 (HHV-6) and human herpesvirus 7 (HHV-7)

The mean age of patients was 25 ± 18 years, 75% of whom were female (Table 2). Nine (33%) patients with ALF from previously unrecognized viral pathogens were positive for betaherpesviruses, specifically, six (22%) HHV-6, one (3%) HCMV and two (7%) dual infections (one with HHV-7/HHV-6, and the other with HHV-7/ HCMV). Interestingly, HHV-7 infection was detected only in the presence of other betaherpesviruses (Table 2). All samples showing positivity in singleplex were also positive in the multiplex assay (Fig. 1).

**Table 2:** Cycle threshold of qPCR specificity analysis for detection and quantification of betaherpesviruses

Target	Cycle Threshold (Ct)
HCMV	35.65
HHV-6	31.12
HHV-7	31.46
HSV-1	UND^a^
HSV-2	UND^a^
EBV	UND^a^
Negative Control	UND^a^

^a^ Undetected

### Specificity analysis

In this experiment, betaherpesviruses were detected but no other herpesviruses tested, clearly indicating specificity for betaherpesvirus targets (Table 3)

**Table 3:** Descriptions and clinical outcomes of ß-herpesvirus populations positive for ALF of unknown etiology

Patient	Sample	Gender	Clinical Outcome	ß-Herpesviruses Positive
FHF-3	Liver	F	Death	HHV-6
FHF – 4	Serum	F	Discharge	HHV-6
FHF – 5	Liver	M	Death	HHV-6/HHV-7
FHF – 6	Serum	F	Discharge	HCMV
FHF – 13	Liver	F	Death	HHV-6
FHF – 14	Serum	M	Discharge	HCMV
FHF – 17	Liver	F	Death	HCMV/HHV-6/HHV-7
FHF – 26	Liver	M	Death	HHV-6
FHF – 27	Liver	F	Discharge	HHV-6

### Reference control

The internal reference control, RNase P, was used with negative samples for betaherpesviruses and samples from healthy patients. The Ct values and RNase P gene quantification remained constant, eliminating the possibility of false negative results (Table 4).

**Table 4:** Evaluation of human RNase P gene as an internal reference control in TaqMan qPCR for betaherpesviruses

CtRNase P (ALF Samples)	±SD	CtRNase P (Controls)	±SD
29.93	0.07	27.55	0.01
25.81	0.11	28.47	0.2
27.06	0	28.46	0.12
22.84	0.33	29.03	0.32

*SD* Standard Deviation

### Detection via Pan herpesvirus PCR and sequencing

Nine positive samples were examined via Pan herpesvirus nested PCR and genome sequencing. In nested PCR using oligonucleotides to amplify the herpesvirus *Dpol* region, all samples previously positive with singleplex and multiplex qPCR were also positive for Pan herpesviruses. Sequencing was performed from Pan herpesvirus-positive samples using the *Dpol* region and all samples were successfully amplified.

### Biochemical analysis of samples

**Fig. 2:**
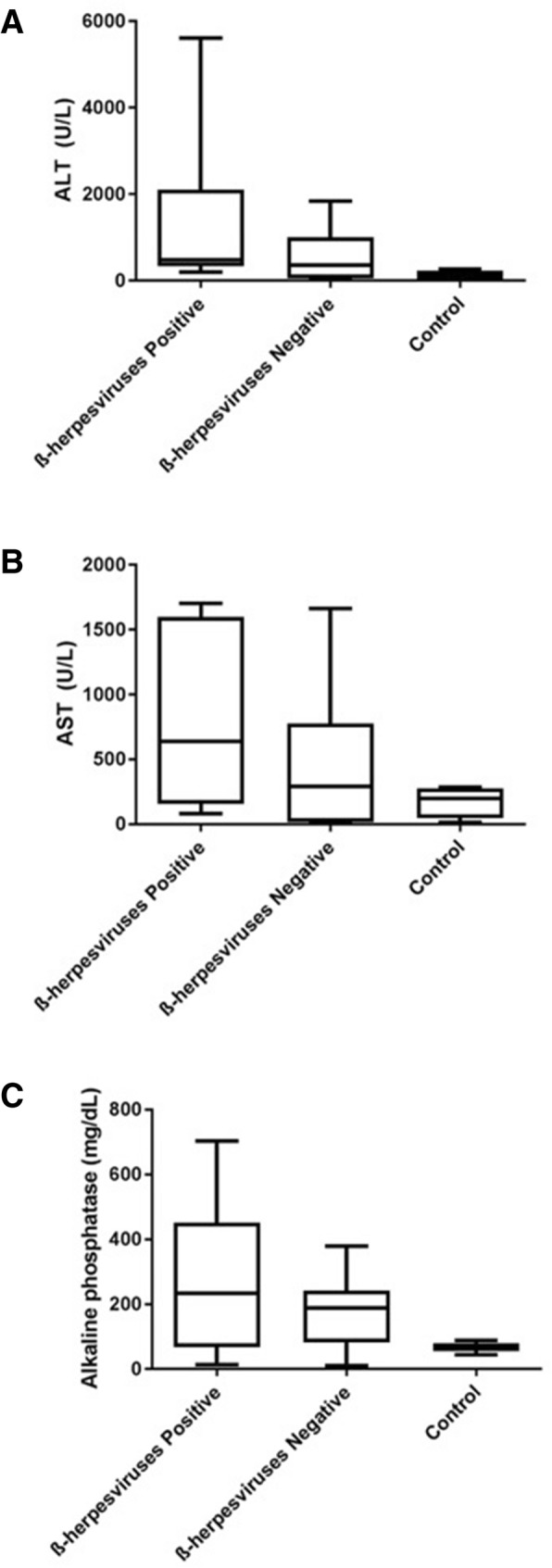
Comparison of alanine transaminase (ALT), aspartate transaminase (AST) and alkaline phosphatase (ALP) levels among healthy control, betaherpesvirus-positive and -negative ALF patient samples

Enzyme evaluation was performed on control samples and betaherpesvirus-positive and -negative ALF samples. In betaherpesvirus-positive ALF samples, alanine transaminase (ALT) and aspartate transaminase (AST) enzyme values were > 1000 U/L. Additionally, hepatic alkaline phosphatase (ALP) presented elevated levels reaching 200 mg/dL. The average of INR value was 5.25. In comparison, the above values were lower in betaherpesvirus-negative and control samples (Fig. 2).

## Discussion

Poor prognosis of hepatitis evolving from herpesvirus infection is associated with late diagnosis and consequently, delayed initiation of antiviral therapy. One major reason for delayed diagnosis is the lack of specific symptoms. Typical mucocutaneous lesions are absent in half of the patients developing hepatitis [3, 17], highlighting the need for early diagnosis for optimizing therapy. In this study, 23.6% samples from ALF patients displayed positivity for at least one of the betaherpesviruses. Our collective results support the utility of qPCR in detecting and quantifying HCMV, HHV-6 and HHV-7 in ALF samples of unknown etiology.

Recent years have seen an increased use of real-time PCR assays to simplify and reduce performance time and quantify virus in a single reaction [20]. In our experiments, RNase P as an internal reference control demonstrated the absence of false-negative results related to detection and quantification of betaherpesviruses in ALF samples with no defined etiology, supporting the accuracy of the results. In addition, multiplex qPCR was specific for betaherpesviruses, since we observed no amplification of other viruses of the Herpesviridae family examined, including HSV-1, HSV-2 and EBV. This result confirmed the specificity of the regions used for betaherpesvirus amplification as well as exclusion of false-positive results potentially caused by the interference of substances or organisms with similar sequences [14]. In the multiplex assays used in this study three fluorescence dye combinations were used to label the probes (FAM, VIC and NED). It allowed that the detection of three types of betaherpesvirus (CMV, HHV-6 and HHV-7). Furthermore, all samples identified as positive via qPCR were confirmed using Pan herpesvirus PCR and sequencing analyses. However, the multiplex is sufficient for screening ALF patients. Although hepatitis viruses are the most common cause of ALF, herpesviruses have also been associated with incidence of hepatitis [16, 21, 22].

In this study, three samples were detected as positive for HCMV. This virus has been reported in cases of liver transplantation in addition to being associated with induction of hepatitis [23, 24]. The association of HCMV replication with ALF suggests a condition that increases the occurrence of HCMV infection after transplantation [24], leading to a requirement for prophylactic antiviral treatment to prevent HCMV infection in transplant recipients [25]. To date, studies have revealed that HCMV is not the main cause of ALF but rather the most common hepatic manifestation in immunocompromised patients [26].

Higher presence of HHV-6 was observed in the ALF samples tested. Among the samples positive for betaherpesviruses, 66% were HHV-6-positive. The greater positivity for HHV-6 corroborates with previous results showing that HHV-6 is more prevalent and pathogenic in ALF cases [9, 13]. In another study, ALF was associated with primary HHV-6 infection in one child [27]. Several other HHV-6-associated cases have been reported, mainly in infants [8, 12]. Earlier cases of ALF have similarly been attributed to HHV-6, although the virus is not yet classified as a causative agent of liver disease [4]. In a previously study, was showed that 12 of 15 patients with disease from an unknown cause and four of 17 patients with ALF from known causes contained HHV-6 antigens in the explanted liver [8], along with a Brazilian case of ALF with HHV-6B [9].

Notably, positivity for HHV-7 was only detected in the presence of other betaherpesviruses. Two cases of dual infection (HHV-7/HHV-6 and HHV-7/HCMV) were identified within our patient population. Co-infection between roseoloviruses (HHV-6 and HHV-7) has been described in a previous studies on post-transplant patient liver samples [10]. Although, in this study was not possible demonstrate that HHV-6 precursor infection can induce subsequent HHV-7 infection, this co-infection should be better investigated.

Some evidence suggest that the molecular mechanisms of viral latency and reactivation are shared among these viruses. HHV-6B is reactivated from latency after coinfection with HHV-7 [28], and HCMV disease is frequently associated with concurrent HHV-6 and HHV-7 reactivation in transplant patients [29, 30]. Studies have suggested an association between HHV-6 and HHV-7 reactivation and increased risk of CMV disease among kidney and liver recipients [31–33], but it is unclear if HHV-6 and 7 infection truly potentiates CMV disease or if the presence of these viruses represent more immunosuppression and attendant risk of CMV [34].

Evaluation of alanine transaminase (ALT) and aspartate transaminase (AST) enzyme levels disclosed values > 1000 U/L in ALF samples positive for betaherpesviruses. Destruction or injury of liver cells releases these enzymes into the circulation [35] and injury-related levels can reach up to 100 times the upper limit reference values of ALT and AST in cases of ALF. Values higher than 1000 U/L are usually reported in cases of viral hepatitis or hepatitis induced by drugs [35, 36]. Another biochemical parameter of liver function determined was INR of 5.25. The elevation time of coagulation represents low prothrombin production by hepatocytes.

Alkaline phosphatase (ALP) is not a hepatocyte-specific marker. However, ALF patients presented elevated levels of ALP of up to 200 mg/dL. As determined previously, ALP displays small elevations in normal levels in cases of infectious hepatitis [35]. However, biochemical results disclosed no significant differences in values in hepatitis cases due to the limited sampling populations analyzed.

In our study, 55.5% positive patients died, consistent with earlier literature showing that ALF often progresses to multiorgan failure, resulting in death or transplantation in 57% cases [37] A significant proportion (12%) of ALF cases worldwide are of unknown origin, often termed indeterminate [37]. In total ~ 2000 cases per year of liver failure are diagnosed in the United States [38], averaging ~ 1 to 6 cases per million worldwide. In Brazil, 2% liver transplants are attributable to hepatic failure [39]. In the absence of information on etiology of the disease, late therapy is initiated in many cases, leading to ineffective treatment and ultimately death.

The incidence of betaherpesviruses (HCMV, HHV-6 and HHV-7) is common worldwide. HCMV has a prevalence of 70–80% in adults [7]. HHV-6 and HHV-7 are ubiquitous, with higher prevalence in adults of up to 90% [40]. The high prevalence of betaherpesviruses can favor opportunistic infections, as seen in ALF, potentially due to virus reactivation as a result of decreased host immune system function [41, 42]. Betaherpesviruses-associated diseases may be decrease by prophylactic treatments with antiviral drugs [43]. Therefore, considering the interval among the appearance of early specific symptoms and the development of ALF, establishing the presence of active infection by herpesvirus in ALF patients is important to quickly start therapy with antiviral for prevent extensive liver damage.

## Conclusion

Although we have not showed a clear association with betaherpesvirus and ALF, this is considered a rare cause. However, several cases of hepatitis highlighting herpesviruses as the most probable cause of this disease have been described in the literature. In keeping with previous findings, data from our study found betaherpesviruses in ALF cases and co-infection of HHV-7 with HHV-6 and CMV. Therefore, diagnosis should be made with caution after excluding classic hepatotropic viruses as another potential cause of disease. Evaluation of betaherpesviruses should be included for differential diagnosis in cases of ALF in order to improve specificity of treatment and minimize patient death. Thus, the multiplex qPCR used in this study simultaneously detected betaherpesviruses and may be useful for rapid and differential diagnosis in cases of ALF with unknown ethiolog.

## Data Availability

The datasets used and/or analysed during the current study are available from the corresponding author on reasonable request.

## Abbreviations

ALF: Acute liver failure
ALP: Alkaline phosphatase
ALT: Alanine transaminase
AST: Aspartate transaminase
EBV: Epstein-barr-virus
HCMV: Human Citomegalovirus
HHV-6: Human Herpes Virus 6
HHV-7: Human Herpes Virus 7
HSV-1: Herpes Simplex virus 1
PCR: Polymerase chain reaction
qPCR: Polymerase chain reaction quantitative
